# An open-label randomized clinical trial to evaluate the efficacy of everolimus *versus* tacrolimus in triple maintenance immunosuppressive therapy for kidney transplant patients

**DOI:** 10.1590/1414-431X20209369

**Published:** 2021-03-03

**Authors:** B.P.S. Assis, M.F. Lasmar, R.A. Fabreti-Oliveira, S.A. Araujo, J. Oliveira, D.C. Wanderley, E. Nascimento

**Affiliations:** 1 Hospital Universitário da Faculdade de Ciências Médicas, Belo Horizonte, MG, Brasil; 2 Faculdade de Ciências Médicas, Belo Horizonte, MG, Brasil; 3 IMUNOLAB Ltda - Laboratório de Histocompatibilidade, Belo Horizonte, MG, Brasil; 4 Instituto de Nefropatologia, Belo Horizonte, MG, Brasil

**Keywords:** Kidney transplantation, Clinical trial, Everolimus, Tacrolimus, Graft survival

## Abstract

Tacrolimus (TAC), a calcineurin inhibitor, and everolimus (EVL), an mTOR inhibitor, have been used as immunosuppressive (ISS) drugs in post-kidney transplantation therapy. The objective of this study was to compare the efficacy of EVL *vs* TAC in the ISS maintenance triple therapy. Ninety-seven kidney transplant patients, who received triple maintenance therapy with TAC, mycophenolate mofetil (MMF), and methyl prednisone (PRED), were evaluated. After four months of post-kidney transplant therapy, 30 patients enrolled in a randomized controlled clinical trial, in which 16 patients received TAC+MMF+PRED (cohort 1), and 14 patients switched to EVL+MMF+PRED (cohort 2). The patients were followed-up for 36 months. Two patients from cohort 1 lost their grafts after one year due to non-adherence. Two patients from cohort 2 had intolerance to mTOR inhibitors and were switched back to TAC from EVL. One case (6.25%) in cohort 1 and three cases (21.43%) in cohort 2 of acute T-cell-mediated rejection was observed. Antibody-mediated acute rejection (ABMAR) was observed in four patients (25.0%) in cohort 1, and antibody-mediated chronic rejection (ABMCR) was observed in two patients (12.50%). One patient from cohort 2 lost the graft after 15 months due to polyomavirus infection. The graft survival rate was 87.50% in cohort 1 and 92.86% in cohort 2. This clinical trial showed that the EVL+MMF+PRED triple maintenance therapy was efficacious compared with TAC during 32 months of follow-up. However, further studies are needed to confirm the efficacy of this regimen for long-term graft survival.

## Introduction

Tacrolimus (TAC), a calcineurin inhibitor (CNI), is an immunosuppressive drug associated with a substantial risk of nephrotoxicity. After kidney transplantation (KT), TAC is administered to inhibit cytokine (IL-2, IL-5, and IFN-γ) production and downregulate T cell activation. It is one of the most effective and widely used immunosuppressive drugs to prevent rejection and increase graft survival (1,2). Despite its effectiveness, it can cause acute and chronic nephrotoxicity following allograft dysfunction (3). Therefore, research has focused on other drugs that are less associated with nephrotoxicity or have minimal adverse effects in patients (4–7). The premature discontinuation of a CNI therapy within 3 months rather than the protocol-recommended 6 months can prevent an increase in creatinine clearance that requires switching to an mTOR inhibitor in the short period for maintenance therapy to improve allograft function (8–11).

Everolimus (EVL) is an mTOR inhibitor drug associated with minor nephrotoxic side effects, which helps to avoid TAC-induced nephrotoxicity (5,9 [Bibr B10],^12^). Different immunosuppressive protocols for preventing graft rejection and adverse effects have been tested to preserve long-term graft survival in association with suitable allograft function and minimal nephrotoxicity, as observed for EVL (13,14). EVL has a half-life of 24 h and inhibits the formation of the mTORC1 protein complex, thereby blocking cell growth and T lymphocyte cell proliferation, which prevents allograft rejection and preserves kidney graft function. To avoid nephrotoxicity, minimal doses of TAC have been used in combination therapy with EVL (6,8). The optimal maintenance immunosuppressive (ISS) therapy in kidney transplantation is not established, but many ISS drugs have been used in different protocol regimens (15–22). However, their long-term use for maintenance therapy have not been evaluated.

The most challenging problem in kidney transplantation is the nephrotoxicity caused by the main ISS drugs, such as cyclosporine A or TAC that have been associated with allograft failure (20–23). On the other hand, the individualized use of EVL for long-term maintenance therapy is required to minimize nephrotoxicity and graft loss. The objective of this study was to assess the efficacy of EVL in triple maintenance therapy compared with the traditional immunosuppressive drug TAC in patients undergoing a 32-month follow-up after KT.

## Material and Methods

### Study population

**Figure 1:**
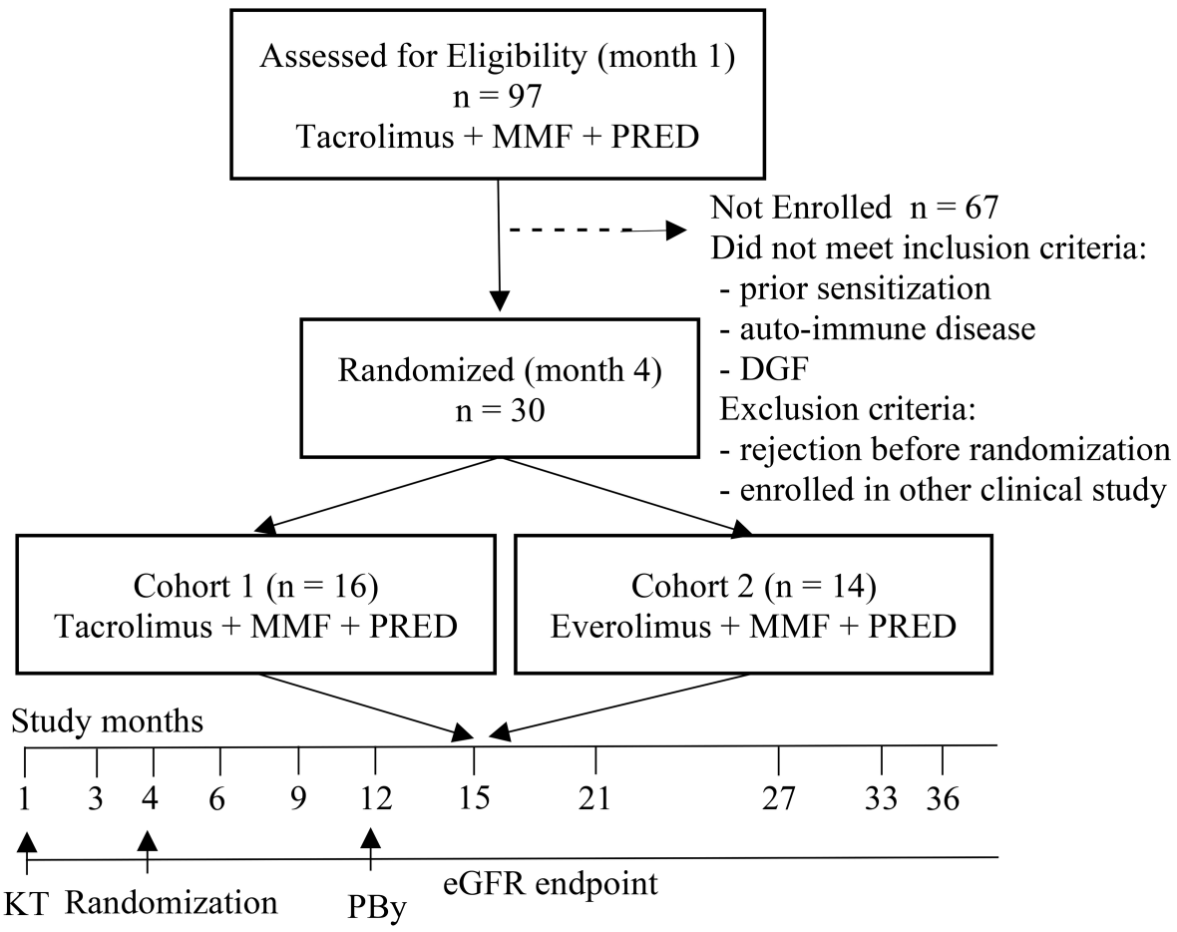
Clinical trial flowchart. MMF: mycophenolate mofetil; PRED: methyl prednisone; PBy: biopsy protocol; KT: kidney transplant; eGFR: estimated glomerular filtration.

The study was conducted using a non-randomized convenience sample of 97 recipients of both genders and between ≥18 and <65 years of age, who received their first KT from living or deceased donors between 2013 and 2015 at the Unit of Kidney Transplantation of the University Hospital of the Faculty of Medical Sciences, Belo Horizonte, Brazil. The inclusion criteria were signing the informed consent form and having completed immunological tests before transplantation, such as human leukocyte antigen (HLA) typing and solid-phase immunoassay-single antigen beads (SPI-SAB) using Luminex platform and crossmatches. Out of the 97 KT subjects, 67 patients were excluded because they were diagnosed with autoimmune disease, had positive SPI-SAB, developed delayed graft function, experienced rejection episodes before randomization, had induction therapy, were enrolled in another clinical trial, or did not follow the hospital ambulatory protocol for KT patients. The estimated glomerular filtration rate (eGFR) was determined 1, 3, 6, 9, 12, 15, 21, 27, 33, and 36 months after KT (Figure 1).

### Immunological evaluation

Presurgical immunological evaluations of the patients and donors were performed by medium resolution for the HLA-A, -B, -C, and -DRB1 loci (One Lambda, USA) using a 100IS fluoroanalyzer (Luminex Inc., USA). The anti-HLA antibody levels were assessed using the SPI-SAB assay (One Lambda) (24). The beads with normalized median fluorescence intensity (MFI) of >500 (i.e., the cutoff value recommended by One Lambda) were considered positive, and crossmatches for B and T lymphocytes (24,25) were performed in the IMUNOLAB, Laboratory of Histocompatibility (Brazil). The diagnosis of rejection, which was graded according to the Banff 2013 and 2015 classification systems (26,27), was performed by the Nephropathology Institute (Brazil). The ethics committee for human research of the Faculty of Medical Sciences of Minas Gerais, Brazil, approved the study protocol and the informed consent forms, which were signed by patients before transplantation under permit CAAE #0054.0.418.000‐10. The study was conducted according to the principles of the Helsinki and Istanbul Declarations. Following the study design, the biopsy protocol was implemented in all patients at the 12-month endpoint and by medical recommendation at any time during the study (Figure 1).

### Triple maintenance and rescue therapy

All KT patients received triple maintenance therapy of 0.25-0.3 mg/kg TAC early after transplantation (Libbs Laboratory, Brazil) with target whole-blood concentrations of 10-12 ng/mL in the first month and 8-10 ng/mL in the following month. Every patient received TAC therapy with concurrent administration of oral mycophenolate mofetil (MMF) (Novartis, Switzerland) at 720 mg twice daily and oral methylprednisolone (PRED) (Meticorten, Sheering-Plough, Brazil) at 0.5 mg/kg in the first 2 months and at 5 mg/day in the following months after transplantation. The 30 patients were randomized four months after KT into two cohorts (C1 and C2). C1 had 16 patients who were maintained by continuous therapy with the same doses of TAC+MMF+PRED, as described above. In the 14 patients assigned to C2, TAC was switched to 1-4 mg/day EVL (Novartis, Switzerland) to achieve target whole-blood concentration of 3-8 ng/mL. The C2 patients were also maintained with MMF+PRED at the same doses as the C1 patients.

All patients were monitored for 36 months after KT (Figure 1) and drug concentrations were measured in the blood at 1, 3, 6, 12, 24, and 36 months after transplantation. These protocols included individualized drug dose adjustments according to blood concentration of the drugs, clinical evaluations, and side effects, such as diarrhea, abdominal pain, weight loss, skin cancer, and infection by cytomegalovirus, polyomavirus, or human papillomavirus. Blood samples for laboratory analysis were collected from enrolled patients 30 min before the next dosing of TAC or EVL.

Patients with clinical symptoms of graft rejection underwent additional biopsy at the hospital when blood samples were collected to perform SPI-SAB. Patients with T-cell-mediated rejection (TCMR) classified according to the Banff criteria (26,27) as cases of borderline IA, IB, and IIA were treated with PRED at 1000 mg/day for 3 days. Patients with corticosteroid-resistant rejection classified as IIB or III were treated with immunotherapy using 7 mg/kg rabbit antibody thymoglobulin (rATG) for 5 to 7 days. The 7 mg/kg of rATG doses were administered based on the number of blood lymphocytes and platelets. Patients were treated and monitored daily using the threshold parameters of >300 lymphocytes/mm^3^ and >5×10^4^ platelets/mm^3^.

The rATG concentration was reduced to 1 mg/kg when the cell count was <300 cells/mm^3^, and patients with <5×10^4^ platelets/mm^3^ received 0.5 mg/kg. The rATG treatment was temporarily suspended or interrupted if severe adverse events, such as anaphylaxis, pulmonary edema, malignancies, or virus infections, were detected clinically or through laboratory testing (28). Patients with antibody-mediated rejection (AMR), who were diagnosed by biopsy and tested positive for complement fraction (C4d+) and donor-specific antibody (DSA+), were treated using a combination of plasmapheresis, 720 mg MMF twice a day, and immunotherapy with rATG from 5 to 7 days, adjusted according to the minimum leukocyte and platelet levels, as described above. The reversal of rejection was defined as a change in the serum creatinine level or eGFR 20 days after rescue therapy. The Brazilian Public Health System provided all maintenance treatment with immunosuppressive drugs.

Drug concentrations were assessed after transplantation for each patient weekly to obtain a target whole-blood level in the first month, every 15 days in the 2nd and 3rd months, monthly for 4 to 12 months, every two months for the second year, every three months for the third year, and each semester in the following years as described in the methodology, and according to previous studies carried out by our research group as published by Lasmar et al. (28).

If the participants presented any adverse events, they would be referred and followed-up for appropriate medical treatment by a team of doctors from University Hospital of the Faculty of Medical Sciences of Minas Gerais. Such treatment would be paid for by the Brazilian Public Health System and the Hospital. Under no circumstances could the treatment be paid for by study participants.

### Statistical analysis

Statistical analysis was performed using SPSS software version 18.0 (IBM, USA), and the Shapiro Wilk normality test was used for all continuous numerical variables. The statistical power of the study was 80%, and differences were considered statistically significant if the P value was <0.05. The means of variables with normal distributions were compared using Student's *t-*test, whereas the variables with non-normal distributions were compared using the Mann-Whitney non-parametric test. Pearson's chi-squared test, Fisher's exact test, and likelihood ratio tests were also used to compare categorical variables. Boxplots were used to graphically visualize the median variations in eGFR during the study period. Graft survival analysis was performed using the Kaplan-Meier method and log-rank tests for group comparisons.

## Results

Patients' sex, age, blood group, etiology of chronic kidney disease, donor age, cold ischemia time, and donor type did not differ significantly between the two cohorts (P>0.05). In contrast, the renal replacement therapy type differed significantly (P=0.033) (Table 1). The analysis of the genetic HLA compatibility between patients and their donors showed that most of them had 1 to 3 HLA mismatches (68.75% in C1 and 57.14% in C2) (Table 1). No statistical differences were observed in the levels of hemoglobin, cholesterol, and hyperlipidemia among patients in both cohorts (Table 2), and no case of nephrotoxicity was observed in the biopsy of patients treated with either TAC or EVL.

**Table 1:** Demographic and clinical data of kidney transplant patients treated with tacrolimus (TAC, cohort 1) and everolimus (EVL, cohort 2).

Variables	Cohort 1 (TAC) (n=16)	Cohort 2 (EVL) (n=14)	P-value
Gender			
Male	13 (92.86%)	9 (64.29%)	0.417
Female	3 (21.43%)	5 (35.71%)	
Recipient age (years)	41.94±13.58	47.79±14.87	0.270
Blood groups			
O	7 (50.00%)	8 (57.14%)	0.429
A	6 (42.86%)	4 (28.57%)	
B	3 (21.43%)	1 (7.14%)	
AB	0 (0.00%)	1 (7.14%)	
CKD etiology			
Hypertensive nephropathy	1 (7.14%)	1 (7.14%)	0.480
Diabetes	2 (14.29%)	1 (7.14%)	
Glomerulopathy	0	1 (7.14%)	
Autosomal polycystic kidney disease	1 (7.14%)	3 (21.43%)	
Renal multicystic disease	1 (7.14%)	0	
Undetermined	11 (78.57%)	8 (57.14%)	
Type of RRT			
Hemodialysis	14 (100.00%)	10 (71.43%)	0.033
Peritoneal dialysis	0	2 (14.29%)	
Preemptive	0	2 (14.29%)	
Time in RRT (months)	19.50 (1-75)	21.50 (0-76)	0.429
Donor			
LD	12 (85.71%)	8 (57.14%)	0.442
DD	4 (28.57%)	6 (42.86%)	
HLA MM			
1 to 3 MM	11 (78.57%)	8 (57.14%)	0.510
4 to 6 MM	5 (35.71%)	6 (42.86%)	
Donor age (years)	39.25±11.03	37.43±11.49	0.662
DD cold ischemia time (h)	12.13±3.66	9.68±7.59	0.590

Data are reported as means±SD, number (%), or median and interquartile range (Student's *t-*test, Mann-Whitney and Pearson's chi-squared test). CKD: chronic kidney disease; RRT: renal replacement therapy; LD: living donor; DD: deceased donor; MM: mismatch.

**Table 2:** Outcome by clinical protocol of kidney transplant patients treated with tacrolimus (TAC, cohort 1) and everolimus (EVL, cohort 2).

Variables	Cohort 1 (TAC) n=16	Cohort 2 (EVL) n=14	P-value
Hemoglobin (g/dL)			
Hb 1-month	10.88±2.15	11.41±2.24	0.512
Hb 6-months	13.43±2.48	13.46±1.39	0.973
Hb 1-year	13.84±2.82	13.78±1.52	0.945
Hb 3-years	13.87±1.39	13.34±2.50	0.528
Cholesterol (mg/dL)			
Total pre-KT	164.22±46.21	171.10±51.22	0.890
LDL pre-KT	93.51±35.02	104.66±42.56	0.453
HDL pre-KT	34.03±6.87	38.02±9.31	0.204
Total 4-months	167.56±29.45	179.30±43.90	0.508
LDL 4-months	90.63±27.76	115.10±26.29	0.074
HDL 4-months	36.56±6.29	38.25±10.97	0.690
Total 1-year	200.73±93.14	200.64±52.04	0.998
LDL 1-year	105.36±52.74	139.60±46.61	0.133
HDL 1-year	40.55±10.33	40.10±12.22	0.929
Total 3-years	186.70±37.48	198.50±29.09	0.442
LDL 3-years	100.39±44.64	119.09±30.72	0.306
HDL 3-years	45.77±7.55	45.41±8.85	0.925

Data are reported as means±SD (Student's *t-*test). KT: kidney transplant; LDL: low density lipoproteins; HDL: high density lipoproteins*;* Hb: hemoglobin.

In C1, T-cell-mediated (TCM) acute rejection (TCMAR) was observed in one (6.25%) patient and TCM chronic rejection (TCMCR) in three (18.75%) patients. In the same cohort, four (25.0%) patients had antibody-mediated acute rejection (ABMAR), and two (12.50%) patients had antibody-mediated chronic rejection (ABMCR). The rejection was resolved in four patients, but the loss of graft was observed in two (12.50%) patients, one with ABMAR caused by DSA to HLA-A24 (MFI=3186), and the other with ABMCR caused by DSA to HLA-B38 (MFI=3512) (Table 3), due to non-adherence to triple maintenance therapy.

**Table 3:** Outcome and rejection episodes based on the Banff classification of kidney transplant patients treated with tacrolimus (TAC, cohort 1) and everolimus (EVL, cohort 2).

Variables	Cohort 1 (TAC) (n=16)	Cohort 2 (EVL) (n=14)	P-value
Acute lesion	4 (25.00%)	5 (35.71%)	0.694
Chronic lesion	3 (18.75%)	2 (14.29%)	0.998
T-cell-mediated rejection			
Acute	1 (6.25%)	3 (21.43%)	0.586
Chronic	3 (18.75%)	1 (7.14%)	0.602
Loss	0 (0.00%)	0 (0.00%)	-
Antibody-mediated rejection			
Acute	4 (25.00%)	0 (0.00%)	0.103
Chronic	2 (12.50%)	0 (0.00%)	0.485
Loss	2 (12.50%)	1 (7.14%)^a^	0.485
Allograft functioning after 3 years	14 (87.50%)	13 (92.86%)	0.485

Data are reported as number (%) (Student's *t-*test and Pearson's chi-squared test). ^a^Polyomavirus.

No ABMAR or ABMCR was observed in C2 (Table 3). The loss of graft was observed in one patient in C2 after 15 months due to polyomavirus infection. During the first two years of the study, two patients in C2 switched from EVL to TAC in the second year; one patient had urticaria and the other diarrhea side effects.

The blood concentration of immunosuppressants TAC and EVL decreased gradually during the follow-up period of the clinical trial in both cohorts (Table 4).

**Table 4:** Blood concentrations of tacrolimus (TAC, cohort 1) and everolimus (EVL, cohort 2) in kidney transplant patients.

Immunosuppressant drugs(ng/mL)	Cohort 1 (TAC) (n=16)	Cohort 2 (EVL) (n=14)
Tacrolimus per month		
1	11.03±2.50	-
4	5.89±1.35	-
6	5.73±0.87	-
12	5.31±0.78	-
24	5.20±0.42	-
36	5.24±0.28	-
Everolimus per month		
1	-	-
4	-	5.14±2.04
6	-	5.48±1.73
12	-	4.90±1.31
24	-	4.68±0.82
36	-	4.55±0.56

Data are reported as means±SD.

**Figure 2:**
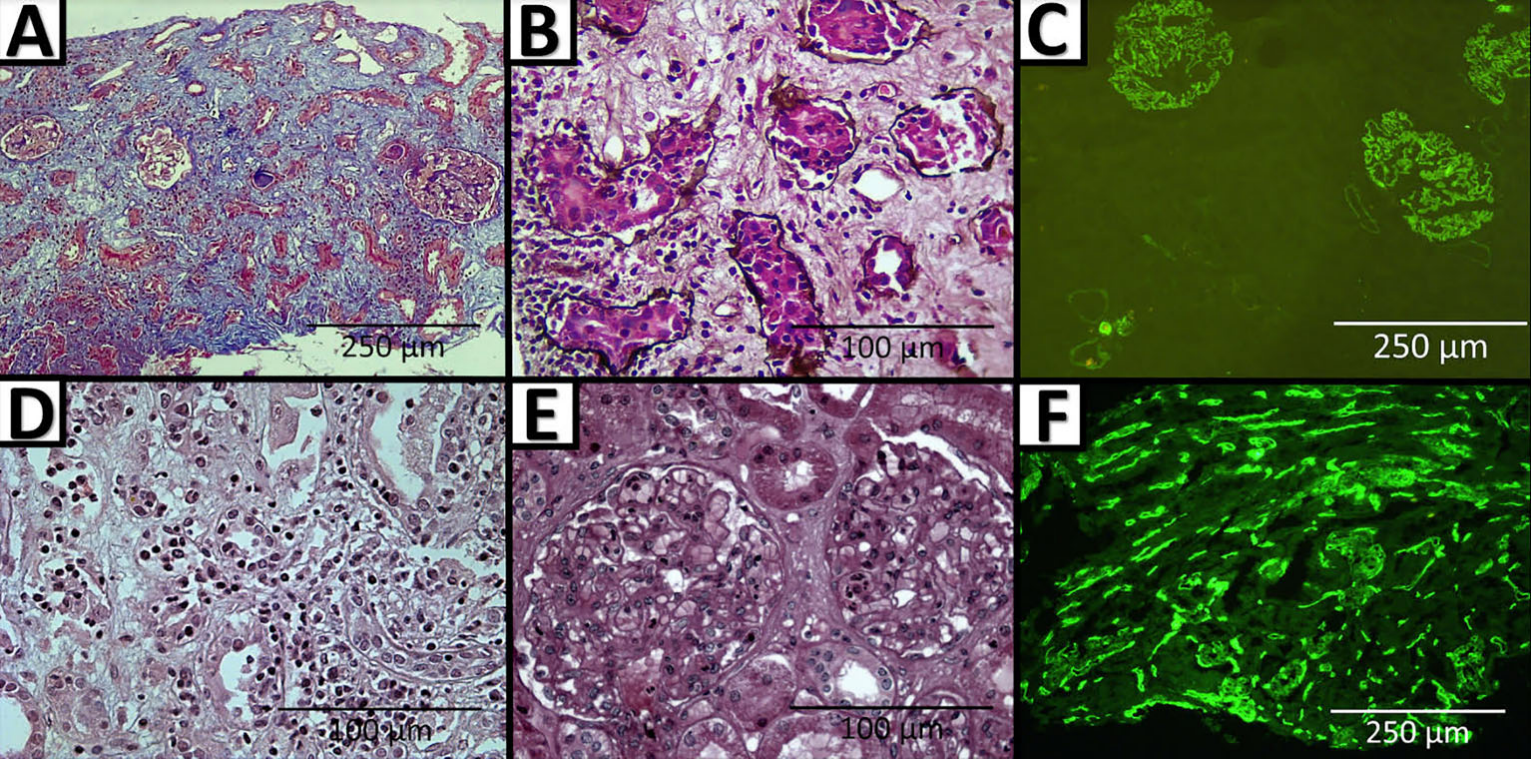
Renal biopsies of two patients from cohort 1 (tacrolimus) who lost their grafts. Patient 1: **A**, inflammation in areas of interstitial fibrosis and tubular atrophy (i-IFTA) (Masson's trichrome stain, 40×, scale bar 250 μm); **B**, chronic cellular rejection due to tubulitis (t3) and interstitial inflammation (i1) (Jones' methenamine silver stain, 400×, scale bar 100 μm); **C**, C4d-negative in peritubular capillaries (C4d immunofluorescence stain (40×, scale bar 250 μm). Patient 2: **D**, diffuse tubulitis (t3), pericapillaritis (ptc1), and interstitial inflammation (i2) (hematoxylin and eosin stain, 400×, scale bar 100 μm); **E**, focal glomerulitis (g1) (hematoxylin and eosin stain, 400×, scale bar 100 μm); **F**, C4d-positive peritubular capillaries (C4d immunofluorescence stain, 40×, scale bar 250 μm).

The renal biopsy results for two patients from C1 who lost their grafts showed that one patient experienced a chronic cellular rejection due to inflammation in areas of interstitial fibrosis and tubular atrophy (i-IFTA), intense tubulitis (t3), and interstitial inflammation (i2) (PAS stain, 40×magnification); C4d was negative per immunohistochemistry stain (at 40×magnification). The second patient experienced active cellular and humoral rejections characterized by diffuse intense tubulitis (t3), interstitial inflammation (i2), focal glomerulitis (g1), and pericapillaritis (ptc1); C4d was positive (C4d2: 10-50%) according to BANFF (25,26) (Figure 2). Among the C2 patients, three (21.43%) patients had TCMAR and one (7.14%) had TCMCR, but maintained good renal function (Table 3).

**Figure 3:**
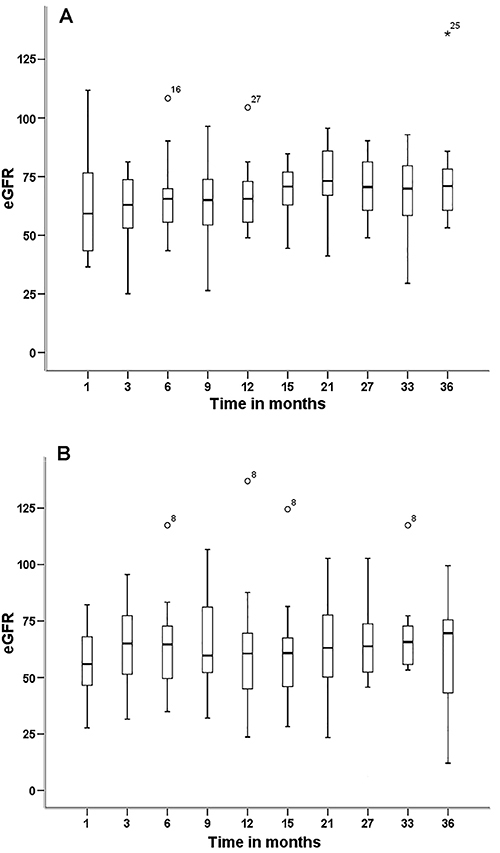
Estimated glomerular filtration rate (eGFR) of kidney transplant patients treated with (**A**) tacrolimus (cohort 1) and (**B**) everolimus (cohort 2) at different time-points (months). Data are reported as medians and interquartile range (Mann-Whitney test).

**Figure 4:**
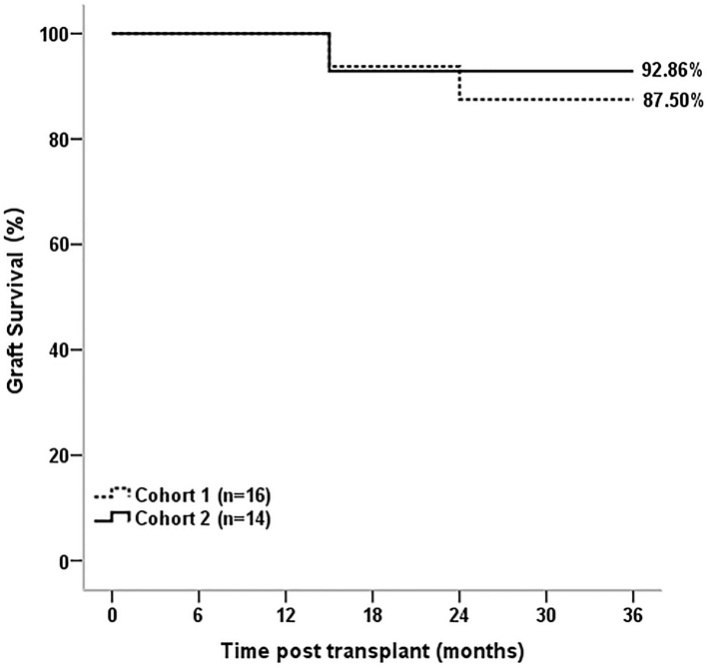
Kaplan-Meier curve of graft survival in kidney transplant patients treated with tacrolimus (cohort 1) and everolimus (cohort 2) (P = 0.217) during 36 months of study.

The mean eGFRs of both cohorts did not differ at any time-point [1 (P=0.528), 3 (P=0.429), 6 (P=0.714), 9 (P=0.647), 12 (P=0.925), 15 (P=0.932), 21 (P=0.739), 27 (0.364), 33 (0.421), and 36 (P=0.177) months] (Figure 3). No case of cytomegalovirus infection was observed. Graft survival was 87.50% in C1 patients (TAC-treated) and 92.86% in C2 patients (EVL-treated) (Figure 4).

## Discussion

The evolution of immunosuppression therapy in renal transplantation has resulted in a decline in acute rejection rates and substantial improvement in kidney graft survival. However, the decrease in rejection incidence is the primary endpoint in most trials evaluating the efficacy of new drugs (29).

The future of EVL in maintenance immunosuppressive therapy in KT patients remains under discussion. Prospective and randomized clinical trials have indicated that the combination of an mTOR inhibitor with MMF is not sufficiently effective in preventing acute rejection during the first year of transplantation (4,23). In a clinical trial with mTOR inhibitors and CNI treatment, inferior outcomes were observed in terms of clinical progression in patients treated with EVL-based therapy (30,31). In this clinical trial, we found that patients in C1 experienced more biopsy-confirmed rejection episodes by TCMR or AMR than patients with graft rejection in C2, indicating a higher immunosuppression efficacy of EVL in triple maintenance therapy during the 32 months after conversion from TAC to EVL.

Our data do not agree with data from other research groups that showed an increase in the risk of rejection in patients who switched from a CNI drug to an mTOR inhibitor (22,23,30,31). Moreover, in our outcomes, the switch from TAC to EVL for maintenance therapy after KT was not associated with a change in the eGFR (P>0.05). Although patient survival rates were similar in both cohorts, some surrogate markers, such as eGFR, and two graft losses were observed in patients undergoing CNI treatment due to immunological causes. Furthermore, one graft loss was observed in a patient receiving the mTOR regimen due to polyomavirus infection. Nevertheless, in a prospective randomized trial with 81 patients treated with sirolimus (an everolimus-like drug) and 84 patients in the tacrolimus group, there was no apparent benefit for the CNI-free regimen (32–34).

Inhibition of the mTOR pathway provides clinical benefit to KT patients, but the mechanism leads to certain side effects, including hyperglycemia and hyperlipidemia (35). Hyperlipidemia was considered an adverse effect of the EVL (11,36), but in this study, used in triple maintenance therapy, hyperlipidemia was not observed and the immunosuppressive efficacy was not inferior compared with TAC. This finding may be due to the sample size and time of outcome. In our study, patient survival at 36 months was similar in both cohorts, but graft survival differed, although the difference was not statistically significant. EVL did not induce changes in the patients' laboratory reference values for hemoglobin, cholesterol, or hyperlipidemia. However, two patients had side effects from the use of the mTOR inhibitor and needed to switch to TAC.

### Conclusion

This open-label clinical trial demonstrated that the EVL+MMF+PRED used in triple maintenance therapy had immunosuppressive efficacy that was not inferior to TAC during 32 months of follow-up. However, further studies are needed to confirm the efficacy of this regimen for long-term graft survival.
